# DASSI: differential architecture search for splice identification from DNA sequences

**DOI:** 10.1186/s13040-021-00237-y

**Published:** 2021-02-15

**Authors:** Shabir Moosa, Prof. Abbes Amira, Dr. Sabri Boughorbel

**Affiliations:** 1 Department of Systems Biology, SIDRA Medicine, Doha, 26999 Qatar; 2 Dept. of Computer Science and Engineering, Qatar University, Doha, 2713 Qatar

**Keywords:** Deep learning, Splice site, Genomics, Neural architecture search, Convolutional neural networks

## Abstract

**Background:**

The data explosion caused by unprecedented advancements in the field of genomics is constantly challenging the conventional methods used in the interpretation of the human genome. The demand for robust algorithms over the recent years has brought huge success in the field of Deep Learning (DL) in solving many difficult tasks in image, speech and natural language processing by automating the manual process of architecture design. This has been fueled through the development of new DL architectures. Yet genomics possesses unique challenges that requires customization and development of new DL models.

**Methods:**

We proposed a new model, DASSI, by adapting a differential architecture search method and applying it to the Splice Site (SS) recognition task on DNA sequences to discover new high-performance convolutional architectures in an automated manner. We evaluated the discovered model against state-of-the-art tools to classify true and false SS in Homo sapiens (Human), Arabidopsis thaliana (Plant), Caenorhabditis elegans (Worm) and Drosophila melanogaster (Fly).

**Results:**

Our experimental evaluation demonstrated that the discovered architecture outperformed baseline models and fixed architectures and showed competitive results against state-of-the-art models used in classification of splice sites. The proposed model - DASSI has a compact architecture and showed very good results on a transfer learning task. The benchmarking experiments of execution time and precision on architecture search and evaluation process showed better performance on recently available GPUs making it feasible to adopt architecture search based methods on large datasets.

**Conclusions:**

We proposed the use of differential architecture search method (DASSI) to perform SS classification on raw DNA sequences, and discovered new neural network models with low number of tunable parameters and competitive performance compared with manually engineered architectures. We have extensively benchmarked DASSI model with other state-of-the-art models and assessed its computational efficiency. The results have shown a high potential of using automated architecture search mechanism for solving various problems in the field of genomics.

## Background

Deep Learning is a class of Machine Learning (ML) algorithms that combines raw inputs into layers of intermediate features. They take raw features from large datasets and use them to create a predictive tool from hidden patterns in the data. They have shown impressive results over existing best-in class ML algorithms across various domains. For the past five years, DL algorithms have revolutionized fields such as high-energy physics [1], computational chemistry [2], dermatology [3]. The off-the-shelf implementation of these algorithms across different fields have produced comparable or higher accuracies than previous state-of-the art methods that required extensive customization over the years.

The advancement of neural networks have demonstrated revolutionizing achievements in the field of image classification, object detection and natural language processing. Designing these neural network architectures requires computational resources and significant efforts from human experts in DL through trial and error. Over the recent years, there has been a paradigm shift from feature designing to architecture designing in the field of image classification and natural language processing [4–7] to develop algorithmic solutions for automating the manual process of architecture design using Neural Architecture Search (NAS) methods. They have provided promising results in designing models better than human designed ones on benchmark datasets. The goal in architecture search is to find an optimal architecture from a given search space so that the validation accuracy on a particular task is maximized. NAS has some similarities to program synthesis and inductive programming where a program is searched from examples [8]. Many architecture search algorithms have been proposed such as Reinforcement Learning (RL) [7] which uses a policy gradient algorithm to optimize architecture configurations. This approach is computationally expensive and time consuming as they design and train each network from scratch during the exploration in the search space. Several approaches were proposed for improving the efficiency of NAS such as establishing a particular structure for the search space [4], performance prediction [9] and weight prediction of individual architectures [10] and by a parameter sharing mechanism [11] across multiple architectures. A novel approach of searching the architectures over a continuous domain alternate to searching over a discrete set of child architectures was proposed in [12]. This Differential Architecture Search (DARTS) mechanism has outperformed various other architecture search algorithms by achieving competitive performance over a rich architecture search space by using less computation resources.

In this paper, the DARTS technique used in [12] for image classification tasks was adapted to solve problems in genomics domain. The study was performed on the Splice Site Recognition (SSR) datasets which provides a scope for analysis of the human genome and identification of unknown regions to understand the biochemical processes involved in building and maintaining a human body. We extended the study to evaluate the discovered architectures on four other different species (Arabidopsis thaliana, Caenorhabditis elegans and Drosophila melanogaster).

### Splice site recognition problem

Proteins form an essential component in all living organism and a major biological process that occurs in all living cells is the production of proteins. They play a vital role in the biochemical reactions within cells and in metabolism. The protein coding process called gene expression occurs in two stages: Transcription and Translation. During Transcription, the DNA (Deoxyribonucleic Acid) is synthesized to produce an mRNA (messenger Ribonucleic Acid). The protein coding process occurs in the translation phase where the mRNA sequence is decoded to produce the proteins.

**Fig. 1:**
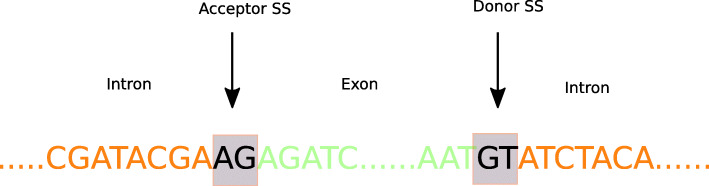
Splice-junction sites on a sequence where the splicing occurs at the exon-intron pairs

Prokaryotic organisms do not have a cell nucleus and their translation stage is relatively simpler. But in eukaryotic organisms the genes are composed of alternated segments of introns and exons. Exons forms the coding regions in a DNA during translation to proteins. The biological significance of intronic regions are not known yet as they do not participate in the protein building process. During translation stage in eukaryotes, the process of splicing occurs where the introns are spliced out from the mRNA molecule. The boundary points where splicing occurs on a gene sequence are called splice junction sites or splice sites as shown in Fig. 1.

Precise identification of Exon-Intron (EI) junctions or donors and Intron-Exon junctions (IE) or acceptors from a sequence is beneficial for advancements in transcriptome research and is a crucial step for fully understanding the gene expression. The accurate detection of splice junctions is challenging because of the high rate of false positives caused by the presence of short canonical splicing patterns [13]. There are currently two different techniques used to solve the splice junction prediction problem: Alignment based techniques and ML based techniques. The sequence alignment-based techniques maps millions of short RNA sequences produced by RNA-seq to the reference genome and then estimate where splicing occur by identifying the adjacent exon locations. The existing alignment based techniques such as SpliceMap [14] and TopHat [15] detects only canonical SS while missing the non-canonical sites which are required for accurate junction prediction. The ML based techniques can predict non-canonical sites as well by appropriate training. Different ML approaches has been used such as Support Vector Machines (SVM) [16–21] Random Forest (RF) [22–24], Decision Trees (DT) [25], Naïve Bayesian (NB) [26], Markov Model [27] and AdaBoost [28–30] to identify splice or non-splice sites. Among them, SVM models have been used very often due to their capability to handle high-dimensional datasets. However, certain kernel and penalty parameters in SVM require extensive tuning which is time consuming. The effectiveness of all these approaches also depends on the feature engineering technique used which is a major initial step in solving a classification problem. Many feature engineering techniques have been proposed for feature construction directly from the DNA sequence,such as the MM1 (1-order Markov model) in [16] for feature construction from splice site sequences and using the SVM for prediction. In [22] and [17] features were constructed based different statistical approach with automated feature extraction was proposed in [26] for prediction of splice sites. A length-variable Markov Model (LVMM) which produced higher accuracy with low time cost was discussed by [27]. A hybrid algorithm of AdaBoost classifier was proposed in [28] which provided an improvement in performance compared to the other approaches. The efficacy of all these approaches is based on the feature extraction step which is often a tedious task that is performed by domain experts. Manual operation of feature extraction often leads to incomplete representation or one-high dimensional feature space which will cause problems in the machine learning process. The challenges involved in performing manual feature extraction and model training led to development of models using Artificial Neural Network (ANN) [31, 32] that performed automated feature representation. Many DL architectures were used and developed for splice site prediction based on CNN [33–37], RNN [13, 38], Restricted Boltzmann Machines (RBM) [39], Autoencoders [40, 41] and Deep Belief Networks [39]. Although these DL architectures have removed the burden of manual feature extraction, they are still time consuming to train and a much deeper knowledge on SS associated functions and evolution has been strongly urged. In general, the existing methods still undergoes the manual effort in designing architectures which needs a lot of expert domain knowledge and is time consuming.

## Methods

**Fig. 2:**
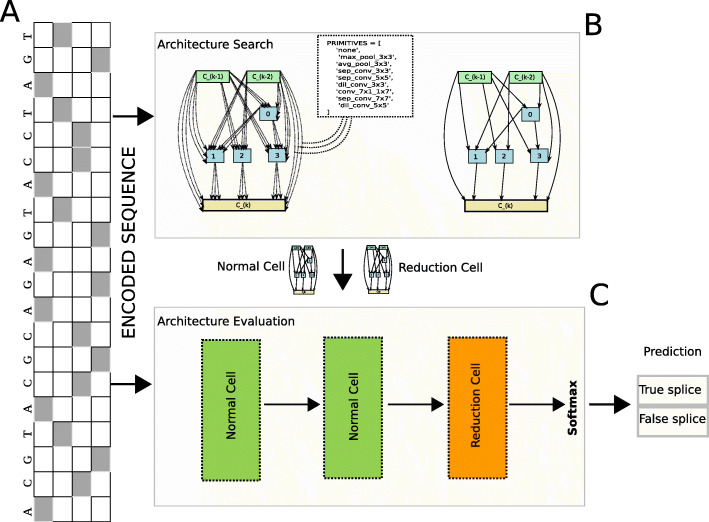
An overview of proposed methodology: It takes the DNA sequence represented as a 2D embedded input (panel **a**) for the Architecture Search(panel **b**) and Architecture evaluation(panel **c**) process

Our model makes use of deep CNN to distinguish features between true and false splice junctions. CNN architectures have shown better performance in learning features that classify true SS from false ones. Figure 2 shows an overview of the proposed methodology and representation of the sequence data. The method consists of two stages: Architecture Search and Architecture Evaluation as shown in Figs. 2b and c. In the first stage, architecture search using DARTS was performed to discover the best model and the second stage validates the discovered model on a held out unseen data. The model gains from the information present in the genomic sequence of the candidate splice junction to accurately classify whether the sequence corresponds to a true splice junction or not.

### Datasets

We have used two datasets for experimental analysis, each comprising of true and false acceptor and donor sequences from four species (Homo sapiens, Arabidopsis thaliana, Caenorhabditis elegans and Drosophila melanogaster). The first dataset *DSS*_1_ [42] has a sequence length of 141 nucleotides and a subset of false SS sequences were randomly extracted to match the number of true SS sequences inorder to avoid the class imbalance problem. The second dataset *DSS*_2_ [35] is a balanced dataset with equal number of true and false SS sequences each having a sequence length of 602 nucleotides. *DSS*_1_ and *DSS*_2_ datasets were used for performance comparison with state-of-the-art models. We used the acceptor sequences of homo sapiens from *DSS*_1_ for computational efficiency evaluation as well as for comparison with generic baseline models. The underlying problem posed in these datasets is to classify, given a sequence of DNA, as a true splice or a false splice sequence. The splice junctions are locations in a DNA sequence where ‘superfluous’ DNA is removed during protein creation process. The beginning and end of an exon is determined by the splice-acceptor and splice-donor sequences present. In this study, the prediction of splice junctions are performed using the given annotated DNA sequences with true acceptor SS and true donor SS sequences as well as false acceptor SS and false donor SS. Table 1 shows the number of true and false SS of each organism used in the experimental evaluations. The datasets were randomly split into three sets into train, validation and test datasets respectively of size 0.8, 0.1, 0.1.

**Table 1:** Description of *DSS*_1_ and *DSS*_2_ datasets

Type	Organism	DSS1	DSS2
Acceptor SS			
	Homo sapiens (Human)	158,217 (True)	248,150 (True)
		158,217 (False)	248,150 (False)
	Arabidopsis thaliana (Plant)	76,871 (True)	112,318 (True)
		76,871 (False)	112,318 (False)
	Caenorhabditis elegans (Worm)	64,838 (True)	77,763 (True)
		64,838 (False)	77,763 (False)
	Drosophila melanogaster (Fly)	29,501 (True)	28,703 (True)
		29,501 (False)	28,703(False)
Donor SS			
	Homo sapiens (Human)	160,601 (True)	250,400 (True)
		160,601 (False)	250,400 (False)
	Arabidopsis thaliana (Plant)	76,659 (True)	110,299 (True)
		76,659 (False)	110,299 (False)
	Caenorhabditis elegans (Worm)	64,844 (True)	77,387 (True)
		64,844 (False)	77,387 (False)
	Drosophila melanogaster (Fly)	29,788 (True)	30,118 (True)
		29,788 (False)	30,118 (False)

### DNA representation

Genome sequence data is biologically described using four types of nucleotide, adenine (A), cytosine (C), guanine(G) and thymine (T). Each of these sequences are converted into numerical representation using one-hot encoding for downstream analysis. However, to shape the input appropriately for the DARTS Convolutional Neural Networks (CNN) model, the DNA sequences are represented as a 2-dimensional tensor. Firstly, one-hot encoding is applied which converts each nucleotide in the DNA sequence of length *n*_*d*_ into a four-dimensional vector and then concatenates each of them to form the complete sequence. The next step is to transform the list of one-hot vectors to a 2-dimensional tensor.

let s *ε* S where S ={A,T,C,G}, then, a sequence (A,C,G,T,A,C) will be encoded into a tuple of 4-D binary vectors as shown in Fig. 2a.

([1,0,0,0],[0,0,1,0],[0,0,0,1],[0,1,0,0],[1,0,0,0],[0,0,1,0])

The encoded sequence is then represented as a two-dimensional tensor of shape (*n*_*d*_ x 4). The final representation of the input to the model will be in the form (batch size x *n*_*d*_ x 4)

### DARTS algorithm

The DARTS method discovers state-of-the art network architectures by formulating the task in a differential manner. The interesting part in this method is that the search space is treated as continuous rather than searching over a discrete set of architectures in the search space.

The cell in the architecture is considered as a Direct Acyclic Graph (DAG) consisting of a set of nodes and edges. Each cell has one output node and two input nodes. Let *N* be the number of nodes and each node represented by *x*^*i*^. Each edge (*i*,*j*) performs an operation represented by *o*^(*i*,*j*)^ that transforms *x*^*i*^. The intermediate nodes are computed based on its predecessors.

1 $$ x^{i}= \sum_{j< i}\left(o^{i,j}x^{j}\right)  $$

The learning of the cell involves learning the operations that transform the input. The goal of the method is to find a cell that forms the building block of the final architecture. Initially the operations on the edges are unknown. Let *O* be the set of operations where each operation is represented as *o*(.) to be applied to *x*^*i*^. The choice of the operation is made in a continuous manner by performing a softmax on all possible operations.

2 $$ \overline {o}^{(i,j)}(x)= \sum_{o\epsilon O}\frac{\exp\left(\alpha_{0}^{(i,j)}\right)}{\sum_{{o'}\epsilon O}\exp\left(\alpha_{0'}^{(i,j)}\right)}o(x)  $$

Here $\alpha _{0}^{(i,j)}$ is a vector with dimension |*O*| that indicates the mixing of operation between a pair of nodes. The architecture search phase jointly performs learning on a set of continuous variables *α*={*α*^(*i*,*j*)^} and the weights (*ω*) within each operation in *O*. The value of *α* and *ω* is obtained through a bi-level optimization algorithm where *α* becomes the higher level variable and *ω* acts as the lower-level variable. The search finds a value of *α* that minimizes the validation loss *L*_*val*_ for that value of *ω* that minimizes the training loss *L*_*train*_.

3 $$ \min_{\alpha}\qquad L_{valid}(\omega^{*}(\alpha),\alpha)  $$

4 $$ s.t\quad \omega^{*}(\alpha)=argmin_{\omega}\quad L_{train}(\omega,\alpha)  $$

This bi-level optimization Algorithm 1 shows the optimization of *ω* and *α* in the respective search spaces through a gradient-based approach. The operation at each edge is replaced by the operation that had the maximum value of *α*.


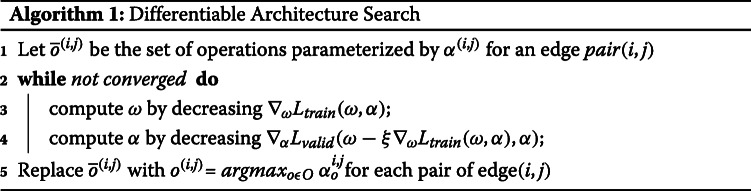


The optimization is performed in the architecture search Algorithm 2 during the training phase and the best discrete architecture *arch*_*final*_ is saved to be evaluated in the architecture evaluation step. The training phase in the architecture evaluation phase has a fixed architecture *arch*_*final*_ and is then trained to obtain the optimal architecture weights. The trained model is evaluated on unseen data in the architecture evaluation phase.


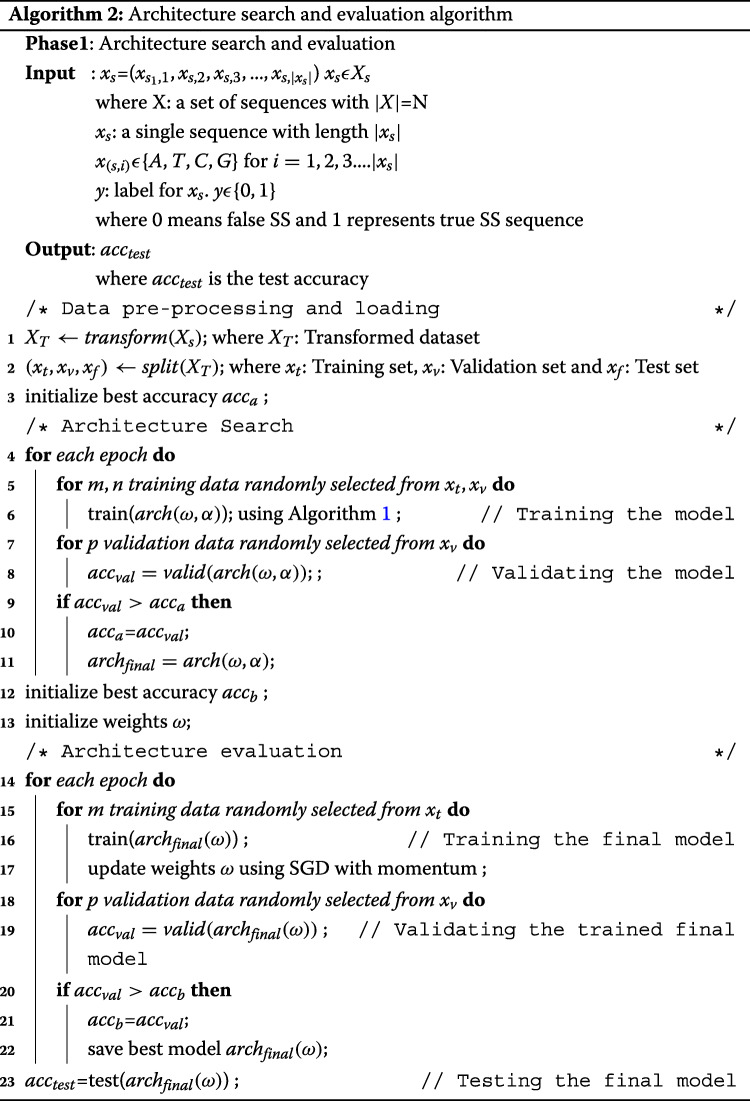


### Experimental setup and implementation

The architecture search and evaluation experiments were performed in the Sidra HPC environment on NVIDIA DGX-1 server. The implementation was done using Pytorch which is an open-source library in python based on torch that supports strong GPU acceleration. The performance evaluation experiments with the state-of-the art architectures were done on datasets *DSS*_1_ and *DSS*_2_ by combining the true and false SS sequences of all species for acceptor and donor separately and tested on unseen dataset of each organism. The baseline model comparisons were performed only on acceptor sequences of homosapiens due to computational overhead.

The computational benchmarking experiments were performed on Intel(R) Xeon(R) x86_64, Quadro K4100M and Tesla V100-SXM2 architectures. The specification of the hardware architectures used is listed in Table 2. The GPUs and CPUs were configured with the same environment as the original experiments. The CPU evaluation was performed by submitting the task as a job to the IBM LSF cluster environment.

**Table 2:** Hardware for Benchmarking

Device Model	Number of available cores	Memory size(in GB)
Tesla V100-SXM2	5120	16
Tesla K40m	2880	12
Quadro K4100M	1152	4
Intel(R) Xeon(R) CPU E5-2670	32	256

#### Architecture search

The following operations were only included from a rich primitive space used in [12] for our search space *O* :3x3 separable convolutions, 5x5 separable convolutions, 7x7 separable convolutions, 3x3 dilated separable convolutions, 1x7 and 7x1 convolution operations, 3x3 max pooling, 3x3 average pooling and a zero operation. A building block of a convolution operation is formed by three steps: Firstly an activation function is Rectifier Linear Unit (RELU) applied and then a convolution operation (CN) is executed and finally, a batch normalization (BN) is performed. This is denoted as ReLU-Conv-BN and we used the same ReLU-Conv-BN order in [12] for performing convolutional operations. Our discovered convolutional cell consisted of 4 nodes, where the output node is result of the depth-wise concatenation of the convolution and pooling layers excluding the input node. The final architecture network was formed by stacking multiple cells together. The architecture consists of two types of convolutional cells called normal cell and reduction cell to make it scalable for any input size. When a feature map is taken as input, the normal cell returns a feature map of same dimension. The reduction cell returns a feature map where the height and width are reduced by a factor of two. The reduction cells are located at 1/3 and 2/3 of the total depth of the architecture. The architecture has a reduction cell in every third cell of the complete architecture. The first and second input nodes of the cell *k* are set to the *k*−2 and *k*−1 cells respectively. A network composed of 3 cells were trained for 50 epochs using DARTS with batch size 100 set for both training and validation. The weights *ω* were optimized using Stochastic Gradient Descent (SGD) with momentum and Adam as the optimizer for architecture variables. The initial learning rate was set as 0.0025 and was gradually decreased to a minimum of 0.001. The rest of the hyperparameters were chosen similar to the original implementation in [12] as shown in Table 3.

**Table 3:** Hyperparameters for architecture search

Hyperparameter	Value
Batch size	100
Number of layers	3
Initial learning rate	0.0003
Architecture learning rate	0.0025
Minimum learning rate.	0.001
Epochs	50
Weight decay rate	0.0003
Loss function	Cross Entropy
Update strategy	SGD with momentum

#### Architecture evaluation

**Fig. 3:**
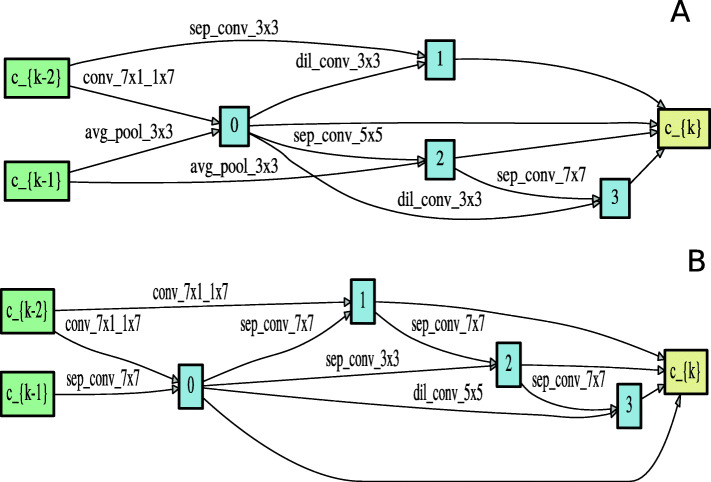
Normal (panel **a**) and Reduction cell (panel **b**) learned on acceptor data

**Fig. 4:**
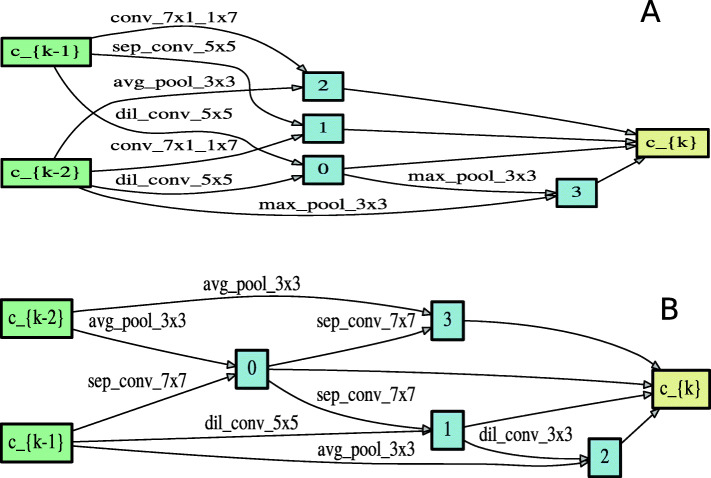
Normal (panel **a**) and Reduction cell (panel **b**) learned on donor data

The best architecture cells for acceptor sequences shown in Fig. 3 and donor sequences shown in Fig. 4 were selected based on the validation performance. The best performing cell was recorded in epoch 46 for acceptor sequences and in epoch 42 for donor sequences. The discovered architecture was trained for 70 epochs with batch size 100. The rest of the hyperparameters were similar to the ones used in the architecture search process. The selected best architecture was evaluated using a held out test datasets of each species. It is important to note that the test set was never used during the architecture search or evaluation processes.

## Results

### Classification results

Firstly, an extensive comparison of DASSI with generic convolutional models based on acceptor sequences of homosapiens from dataset *DSS*_1_ was performed. The architectures of the baseline models are shown in Table 4. The experiments were repeated 10 times based on different random seeds for splitting the training, validation and test sets. Table 5 gives a summary of the results for 5 different performance metrics. Notably, the discovered model outperformed Long Short Term Memory (LSTM) model and baseline CNN with and without embedding in terms of test accuracy, sensitivity, specificity, F-score and AUC score. DASSI achieved better accuracy results over the hybrid and LSTM model with embedding. We also noted that the fixed CNN baseline architectures performed poorly compared to other architectures. After comparing DASSI with generic baselines, two well-known models for splice site identification were considered. The first model, Accurate Splice Prediction (ASP), is a SVM model [42]. The second model is a recent deep learning model named Splice2Deep [35] which achieved state-of-the-art performance for SS identification. Splice2Deep has 6 parallel convolution layers where the output of these layers are concatenated and transformed by a fully connected layer. Three versions of the model were trained: upstream, downstream and global models. The obtained features of the last fully connected layers of these models were concatenated to train a logistic regression classifier for the final prediction. In this study, two DASSI architectures were discovered separately for acceptor and donor, based on sequences from four different species combined together (Homo Sapiens, Arabidopsis thaliana, Caenorhabditis elegans, Drosophila melanogaster).

**Table 4:** Baseline model architectures

Model	Architecture
CNN	FC →*Conv*→*MaxPool*→*FC*→*Dropout*→FC
CNN(emb.)	Emb →*Conv*→*MaxPool*→*FC*→*Dropout*→FC
Hybrid	FC →*Conv*→*Pool*→*LSTM*→FC
LSTM	FC →*LSTM*→*FC*→*Dropout*→FC
LSTM(emb.)	Emb →*FC*→*LSTM*→*FC*→*Dropout*→FC

**Table 5:** Comparison of DASSI Model Performance with baseline models

Model	Accuracy	Sensitivity	Specificity	F-score	AUC score
DASSI _*hs*_	94.15 ±0.1	94.00 ±0.1	95.20 ±0.3	94 ±0.1	98.8 ±0.01
LSTM (emb.)	93.98 ±0.1	95 ±1.0	92.32 ±1.2	93.74 ±0.2	93.66 ±0.2
Hybrid	93.66 ±0.2	94.63 ±0.7	93.33 ±0.6	94.02 ±0.1	93.66 ±0.2
LSTM	86.99 ±14.6	85.26 ±19.8	88.71 ±9.8	85.87 ±16.9	86.99 ±14.6
CNN	64.15 ±0.6	65.27 ±6.0	63.04 ±6.2	64.43 ±2.1	64.10 ±0.5
CNN (emb.)	53.63 ±3.6	95.62 ±6.6	11.63 ±13.7	67.35 ±0.5	53.63 ±3.6

**Fig. 5:**
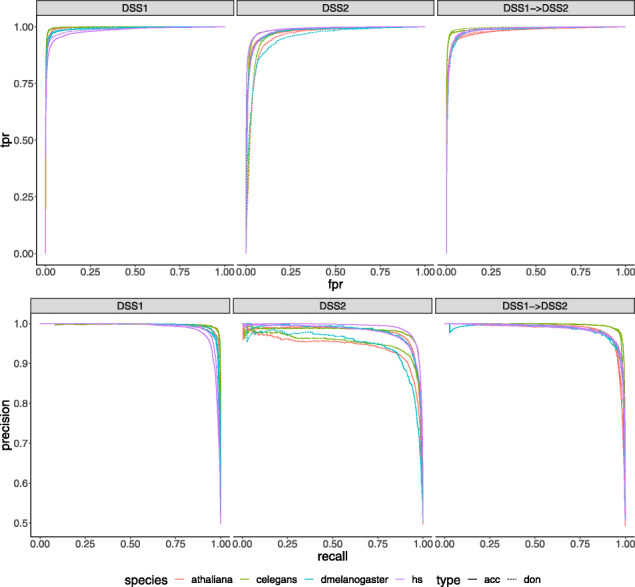
ROC curves (panel **a**) and precision recall curves (panel **b**) of DASSI model for splice site classification on the genome-wide datasets of human, plant, worm and fly

Table 6 presents the comparison of DASSI, ASP and Splice2Deep for the datasets *DSS*_1_ and *DSS*_2_. The comparison is performed for acceptor and donor on each of the four considered species. The AUC (Area Under ROC) and accuracy values in Table 6 for ASP and Splice2Deep were taken from their respective publications. The values that were not reported in the publications have been indicated as missing in the results table. While the results of DASSI model was on par or slightly better than ASP in some cases, it clearly outperformed ASP in the transfer learning scenario as shown in Table 8 and more details on this scenario is provided in the next paragraph. In addition, *DSS*_1_ dataset was down-sampled to keep the number of true and false sequences balanced. Training DASSI on a larger dataset from *DSS*_1_ could potentially improve its results. For *DSS*_2_, while Splice2Deep outperformed DASSI in most cases, the performance gap between the two models is not significantly large when the size of the models represented by the number of tunable parameters is considered. Model sizes are presented in Table 7. DASSI size is much smaller than Deep2Splice and represents only 10% to 20% of the Spice2Deep model size. The performance gap could potentially be closed by increasing the number and size of the primitives in DASSI for the architecture search. Figure 5 displays the ROC and precision-recall curves of DASSI model on all the four different species.

**Table 6:** Performance comparison of models: DASSI, Splice2Deep and ASP-SVM

		DSS1				DSS2			
		ASP-SVM		DASSI		Splice2Deep		DASSI	
Site	Species	AUC	Accuracy	AUC	Accuracy	AUC	Accuracy	AUC	Accuracy
Acceptor	Homo Sapiens	97.86	-	98.22	94.56	98.69	96.91	98.98	95.48
	Arabidopsis thaliana	99.43	-	99.57	97.33	98.31	95.21	97.78	93.78
	Caenorhabditis elegans	99.80	-	99.73	98.18	99.49	98.08	98.42	95.3
	Drosophila melanogaster	99.12	-	99.12	96.57	98.16	94.07	97.78	93.41
Donor	Homo Sapiens	98.63	-	98.7	95.8	99.1	97.38	98	93.33
	Arabidopsis thaliana	99.68	-	99.76	98.24	98.69	95.59	95.51	90.3
	Caenorhabditis elegans	99.82	-	99.8	98.24	99.48	97.68	96.49	92.18
	Drosophila melanogaster	99.51	-	99.44	96.81	99.56	90.52	95.03	88.91

**Table 7:** The number of trainable parameters for Splice2Deep and DASSI. For Splice2Deep the architecture is fixed and the total number of parameters is the sum of the three models: upstream, downsteam and global models [35]. For DASSI, two separate architectures for acceptor and donor were discovered with different number of parameters

	Splice2Deep	DASSI
Acceptor	1.33M	272K
Donor	1.33M	103K

**Table 8:** Performance comparison of DASSI and ASP for transfer learning scenario

		DSS1 → DSS2			
		ASP-SVM		DASSI	
Site	Species	AUC	Accuracy	AUC	Accuracy
Acceptor	Homo Sapiens	80.09	65.82	98.53	94.81
	Arabidopsis thaliana	79.62	71.10	97.96	94.33
	Caenorhabditis elegans	96.72	93.15	99.16	97
	Drosophila melanogaster	96.96	86.63	98.34	94.53
Donor	Homo Sapiens	68.42	60.82	98.65	94.94
	Arabidopsis thaliana	86.94	77.97	98.01	94.63
	Caenorhabditis elegans	84.26	72.63	99.51	97.44
	Drosophila melanogaster	77.98	71.10	98.52	94.32

We further investigated the generalization of DASSI across datasets. We introduced a transfer learning scenario (*DSS*_1_→*DSS*_2_) in which DASSI and ASP (SVM) were trained on *DSS*_1_ and tested on *DSS*_2_. The same test set that was used in previous experiments on *DSS*_2_ was used for the transfer learning approach as well. Since *DSS*_2_ has a sequence length of 602 and the two models were trained on sequences of length 141 from *DSS*_1_, we trimmed the test sequences of *DSS*_2_ such that the dimmer sequence for donor and acceptor is located at the same position as in *DSS*_1_. The pre-trained model of ASP available on github^1^ was used to test on the trimmed sequences of *DSS*_2_. Table 8 provides the performance comparison of DASSI and ASP (SVM) [42] on the trimmed dataset from *DSS*_2_. The performance of the pre-trained ASP was not estimated in this scenario due uncertainty on the parts of the data that was used for training and testing. The accuracy is reported for a default threshold of 0.5. ASP seemed to be not well calibrated for *DSS*_2_ and AUC was chosen as a better metric for comparison. DASSI outperformed ASP in the transfer learning scenario for classifying true and false SS in acceptor and donor sequences on all four species. The transfer learning scenario *DSS*_2_→*DSS*_1_ was not feasible because the models would need to be trained on sequences of length 602 and tested on shorter sequences of 141 and padding the test sequences to match the input size would not be meaningful.

### Computational Performance Results

**Fig. 6:**
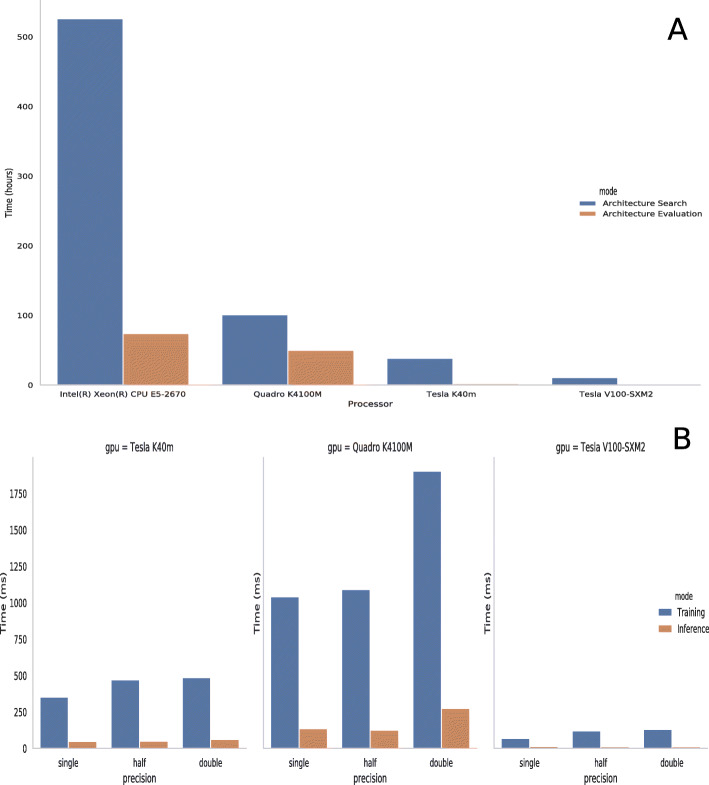
Search and evaluation execution time of the DARTS model on GPU and CPU devices (panel **a**) and training and inference speed of DARTS model on each of the devices (panel **b**)

In addition, the proposed model was benchmarked on GPU systems and CPU for comparison of execution time and precision as shown in Fig. 6

#### Execution time

The execution time for performing architecture search and architecture evaluation were calculated on different device architectures and the results are presented in Table 9. Notably, the most advanced Tesla V100 GPU completed the search in less than 11 hours and the evaluation in half an hour.

**Table 9:** Comparison of Execution Time

Device Model	Architecture Search(hours)	Architecture Evaluation(hours)
Tesla V100-SXM2	10.75	0.5
Tesla K40m	38.5	2
Quadro K4100M	101	50
Intel(R) Xeon(R) CPU E5-2670	526	74

### Precision

The learning and inference speed of the trained model were compared on different GPU architectures as shown in Table 10. The experiments were performed on single precision, half precision, double precision data types. The model was fed with a single batch input of 500 sequences. For training, the time required for 20 forward and backward passes were averaged. In inference, time duration of 20 forward passes were averaged. Five warm up steps were included that was not calculated towards the final results.

**Table 10:** Comparison of Learning and Inference Speed on GPUs

GPU Model	Training			Inference		
	Single	Half	Double	Single	Half	Double
Quadro K4100M	1041.24	1090.38	1902.38	134.97	124.59	274.78
Tesla K40m	352.53	470.72	485.17	48.022	49.38	62.13
Tesla V100-SXM2	68.46	119.55	130.09	13.21	11.02	11.07

## Discussion

Deep Learning is an emerging research topic among the genomics community. Its applications can be revolutionized by introducing high-performance computing methods to analyze datasets in the field of gene therapies, molecular diagnostics and personalized medicine. In the scope of this paper, DASSI - a differential architecture search approach was implemented to solve the splice site classification problem in genomics and to discover new high performance CNN architectures. Despite the slightly lower performance compared with the state-of-the-art models on certain species, the discovered architectures are highly compact and allow very good generalization across datasets and species. This work has aided in bridging the gap between the state-of-the art in DL and its application to genomics. The evaluation results showed that the newly discovered architecture outperformed the fixed baseline DL architectures and showed competitive results against state-of-the-art models. The architecture was also compared alongside the well-known LSTM model and complex hybrid architectures. Furthermore, the discovered architecture was evaluated on multiple CPU and GPU architectures. The total time taken for performing the architecture search and evaluation were determined as well as the floating point instructions per second for single, double and half precision were compared. The computational benchmarking results obtained proved that there is significant improvement in execution time when using advanced GPU architectures.

For all its promises, DL in genomics still possess a number of challenges. The results largely depend on the quality of the data input that are well annotated so that the model can learn to distinguish features and identify patterns. Another challenge is the lack of judgement capability where the technique is able to distinguish from a biologically relevant variation and normal variations. This would require applying further experimental design and controls. The advancements in the field of DL in the field of computer vision and speech recognition has led to new methods being constantly proposed that awaits its application in genomics domain. Furthermore, the availability of quasi-unlimited storage at a reasonable price, the surge in computing power and the lower computational costs will allow these advanced DL techniques to reshape the capabilities of machines to completely understand and interpret the human genome.

## Conclusions

In this study, we applied the differential architecture search technique for performing splice site classification using raw DNA sequences and compared the discovered architectures against well-known fixed baseline architectures and state-of-the art models. As future steps, we plan to further improve the performance of DASSI by including more primitives such as skip connect and additional convolutional operations, thereby widening the architecture search space. This will help to traverse more information to lower layers. The performance will also be evaluated by increasing the number of layers in the architecture. We also plan to perform the search on different species seperately and compare the discovered architectures for different species. DASSI model will further be evaluated against the recent parallel work of Neural Architecture Optimization (NAO) which also performs continuous optimization of architecture space. The study showed that fixed RNN architectures have better results than CNN. It would be interesting to implement DASSI to search for a recurrent cell that can be recursively connected to form a RNN that can be applied for tasks of protein function prediction. In addition, this approach will be tested on additional genomics classification tasks, as it will be highly useful to uncover new insights from the vast available sequencing data.

## Data Availability

The code and data used in the experiments are available at https://github.com/shabirmoosa/DASSI.git

## Abbreviations

CNN: Convolutional Neural Networks
DARTS: Differential Architecture Search
DL: Deep Learning
SS: Splice Sites
DNA: Deoxyribonucleic Acid
LSTM: Long Short Term Memory
ML: Machine Learning
NAS: Neural Architecture Search
NAO: Neural Architecture Optimization
RNN: Recurrent Neural Network
SGD: Stochastic Gradient Descent

## Footnotes

1 https://github.com/ratschlab/ASP
